# Connexin Hemichannel Blockade Is Neuroprotective after Asphyxia in Preterm Fetal Sheep

**DOI:** 10.1371/journal.pone.0096558

**Published:** 2014-05-27

**Authors:** Joanne O. Davidson, Paul P. Drury, Colin R. Green, Louise F. Nicholson, Laura Bennet, Alistair J. Gunn

**Affiliations:** 1 Department of Physiology, The University of Auckland, Auckland, New Zealand; 2 Department of Ophthalmology, The University of Auckland, Auckland, New Zealand; 3 Department of Anatomy with Radiology, The University of Auckland, Auckland, New Zealand; Robert Debre Hospital, France

## Abstract

Asphyxia around the time of preterm birth is associated with neurodevelopmental disability. In this study, we tested the hypothesis that blockade of connexin hemichannels would improve recovery of brain activity and reduce cell loss after asphyxia in preterm fetal sheep. Asphyxia was induced by 25 min of complete umbilical cord occlusion in preterm fetal sheep (103–104 d gestational age). Connexin hemichannels were blocked by intracerebroventricular infusion of mimetic peptide starting 90 min after asphyxia at a concentration of 50 µM/h for one hour followed by 50 µM/24 hour for 24 hours (occlusion-peptide group, n = 6) or vehicle infusion for controls (occlusion-vehicle group, n = 7). Peptide infusion was associated with earlier recovery of electroencephalographic power after asphyxia compared to occlusion-vehicle (*p*<0.05), with reduced neuronal loss in the caudate and putamen (*p*<0.05), but not in the hippocampus. In the intragyral and periventricular white matter, peptide administration was associated with an increase in total oligodendrocyte numbers (*p*<0.05) and immature/mature oligodendrocytes compared to occlusion-vehicle (*p*<0.05), with a significant increase in proliferation (*p*<0.05). Connexin hemichannel blockade was neuroprotective and reduced oligodendrocyte death and improved recovery of oligodendrocyte maturation in preterm fetuses after asphyxia.

## Introduction

Preterm birth occurs in around 7 to 12% of all live births and is associated with a high level of neurodevelopmental disability and cerebral palsy [1]. The predominant injury seen in these infants involves diffuse, non-destructive white-matter lesions in the periventricular and surrounding white matter that is characterized by acute oligodendrocyte cell loss and prolonged arrest of oligodendrocyte lineage maturation [2]. However, there is increasing evidence from post-mortem and imaging studies that acute subcortical neuronal injury also contributes to long-term neurodevelopmental disability [1], [3]. There are currently no clinically proven therapeutic interventions to reduce this brain damage, highlighting the need to better understand the mechanisms underlying the spread of ischemic brain injury in the preterm fetus/neonate.

Hemichannels, or connexons, are half of a gap junction channel that sits in the unopposed membrane of a cell, before the formation of new channels. Opening of connexin hemichannels has been associated with ischemia, as well as oxygen glucose deprivation, metabolic inhibition or low extracellular calcium ion (Ca^2+^) levels [4]–[8]. This may cause disruption of the resting membrane potential, release of cytotoxic levels of ATP [9] and glutamate [10] and uptake of water, leading to cell swelling and death [11], [12]. We have previously shown that blockade of astrocytic connexin 43 hemichannels reduced oligodendrocyte cell loss and seizure activity and improved recovery of brain activity following global cerebral ischemia in the near-term fetal sheep [13]. However, the distribution of injury and particular vulnerability of specific cell types to ischemia varies considerably between the full-term and preterm neonate. Therefore, it is unclear whether connexin hemichannels contribute to the spread of injury following asphyxia in the preterm fetus, when white matter is predominantly populated by oligodendrocyte progenitor cells at a stage when they are most vulnerable to injury [14].

In the present study, we tested the hypothesis that blockade of connexin hemichannels with a specific mimetic peptide after severe asphyxia induced by complete umbilical cord occlusion would reduce loss of oligodendrocytes and neurons and improve recovery of brain activity in 0.7 gestation preterm fetal sheep. At this age, brain development is broadly consistent with 28 to 32 weeks in humans, before the development of cortical myelination [15], [16].

## Materials and Methods

### Ethics Statement

All procedures were approved by the Animal Ethics Committee of The University of Auckland following the New Zealand Animal Welfare Act, and the Code of Ethical Conduct for animals in research established by the Ministry of Primary Industries, Government of New Zealand.

Mean arterial pressure and fetal heart rate were transiently elevated after asphyxia in both groups (Figure 3). Nuchal EMG activity was transiently reduced after asphyxia followed by an increase to above baseline levels in both groups, and was significantly higher in the occlusion-peptide group from 62 to 106 hours (*p*<0.05). There were no significant changes in extradural temperature in either group.

### Fetal Surgery

In brief, 20 time-mated Romney/Suffolk fetal sheep were instrumented using sterile technique at 97–98 days gestation (term is 145). Food, but not water was withdrawn 18 hour before surgery. Ewes were given 5 mL of Streptocin (procaine penicillin (250,000 IU/mL) and dihydrostreptomycin (250 mg/ml, Stockguard Labs Ltd, Hamilton, New Zealand)) intramuscularly for prophylaxis 30 minutes prior to the start of surgery. Anesthesia was induced by i.v. injection of propofol (5 mg/kg; AstraZeneca Limited, Auckland, New Zealand), and general anesthesia maintained using 2–3% isoflurane (Medsource, Ashburton, N.Z.) in O_2_. The depth of anesthesia, maternal heart rate and respiration were constantly monitored by trained anesthetic staff. Ewes received a constant infusion isotonic saline drip (at an infusion rate of approximately 250 mL/h) to maintain fluid balance.

Following a maternal midline abdominal incision and exteriorization of the fetus, both fetal brachial arteries were catheterized with polyvinyl catheters to measure mean arterial blood pressure. An amniotic catheter was secured to the fetal shoulder. ECG electrodes (Cooner Wire Co., Chatsworth, California, USA) were sewn across the fetal chest to record fetal heart rate. And inflatable silicon occluder was placed around the umbilical cord (in vivo Metric, Healdsburg, Ca, USA). Using 7 stranded stainless steel wire (AS633–5SSF; Cooner Wire Co.), two pairs of EEG electrodes (AS633–5SSF; Cooner Wire Co.) were placed on the dura over the parasagittal parietal cortex (5 mm and 10 mm anterior to bregma and 5 mm lateral) and secured with cyanoacrylate glue. A reference electrode was sewn over the occiput. A further two electrodes were sewn in the nuchal muscle to record electromyographic activity as a measure of fetal movement and a reference electrode was sewn over the occiput. A thermistor was placed over the parasagittal dura 30 mm anterior to bregma. An intracerebroventricular catheter was placed into the left lateral ventricle (6 mm anterior and 4 mm lateral to bregma). The uterus was then closed and antibiotics (80 mg Gentamicin, Pharmacia and Upjohn, Rydalmere, New South Wales, Australia) were administered into the amniotic sac. The maternal laparotomy skin incision was infiltrated with a local analgesic, 10 ml 0.5% bupivacaine plus adrenaline (AstraZeneca Ltd., Auckland, New Zealand). All fetal catheters and leads were exteriorized through the maternal flank. The maternal long saphenous vein was catheterized to provide access for post-operative maternal care and euthanasia.

### Post-operative Care

Sheep were housed together in separate metabolic cages with access to food and water *ad libitum*. They were kept in a temperature-controlled room (16±1°C, humidity 50±10%), in a 12 hour light/dark cycle. Antibiotics were administered daily for four days I.V. to the ewe (600 mg benzylpencillin sodium, Novartis Ltd, Auckland, New Zealand, and 80 mg gentamicin, Pharmacia and Upjohn). Fetal catheters were maintained patent by continuous infusion of heparinized saline (20 U/mL at 0.15 mL/h) and the maternal catheter maintained by daily flushing.

### Data Recording

Data recordings began 24 hours before the start of the experiment and continued for the remainder of the experiment. Data were recorded and saved continuously to disk for off-line analysis using custom data acquisition programs (LabView for Windows, National Instruments, Austin, Texas, USA). Arterial blood samples were taken for pre-ductal pH, blood gas, base excess (Ciba-Corning Diagnostics 845 blood gas analyzer and co-oximeter, Massachusetts, USA), glucose and lactate measurements (YSI model 2300, Yellow Springs, Ohio, USA). All fetuses had normal biochemical variables for their gestational ages [17], [18].

Fetal mean arterial blood pressure (MAP, Novatrans II, MX860; Medex Inc., Hilliard, OH, USA), corrected for maternal movement by subtraction of amniotic fluid pressure, fetal heart rate (FHR) derived from the ECG, EEG and EMG were recorded continuously from −24 to 168 hours after umbilical cord occlusion. The blood pressure signal was collected at 64 Hz and low pass filtered at 30 Hz. The nuchal EMG signal was band-pass filtered between 100 Hz and 1 kHz, the signal was then integrated using a time constant of 1 sec. The analogue fetal EEG signal was low pass filtered with the cut-off frequency set with the −3 dB point at 30 Hz, and digitized at 256 Hz (using analogue to digital cards, National Instruments Corp., Austin, TX, USA). The intensity and frequency were derived from the intensity spectrum signal between 0.5 and 20 Hz. For data presentation, the total EEG intensity (power) was normalized by log transformation (dB, 20×log (intensity)), and data from left and right EEG electrodes were averaged. Power in the Delta (0–3.9 Hz), Theta (4–7.9 Hz), Alpha (8–12.9 Hz), and Beta (13–22 Hz) spectral bands was calculated as described [19].

### Experimental Protocols

Experiments were performed at 103–104 d gestation. Fetal asphyxia was induced by rapid inflation of the umbilical cord occluder for 25 minutes with sterile saline of a defined volume known to completely inflate the occluder and totally compress the umbilical cord, as determined in pilot experiments with a Transonic flow probe placed around an umbilical vein [20]. Successful occlusion was confirmed by observation of a rapid onset of bradycardia with a rise in MAP, and by pH and blood gas measurements. If fetal blood pressure fell below 8 mmHg then the occlusion was stopped immediately.

For Cx43 hemichannel blocking, a peptide (H-Val-Asp-Cys-Phe-Leu-Ser-Arg-Pro-Thr-Glu-Lys-Thr-OH (Auspep, Vic, AU)) that mimics the second extracellular loop of Cx43 (‘Peptide 5’ reported in [21]) was infused into the lateral ventricle via the intracerebroventricular catheter attached to an external pump (SS-2222, Harvard Apparatus, Holliston, MA, USA). Vehicle control fetuses received asphyxia followed by infusion of the vehicle (asphyxia-vehicle, n = 7). The asphyxia-peptide group (n = 6) received 50 µmol/kg/h for one hour followed by 50 µmol/kg/24 hours for 24 hours, dissolved in artificial cerebrospinal fluid (aCSF), at a rate of 1 ml/hour for 25 hours starting 90 min after the end of the occlusion. The sham control group received a sham umbilical cord occlusion plus infusion of the vehicle (n = 7). The mimetic peptide was not tested in sham occlusion animals. All animals were killed at seven days with an overdose of sodium pentobarbitone (9 g I.V. to the ewe; Pentobarb 300, Chemstock International, Christchurch, N.Z.).

### Immunocytochemistry

The fetal brains were perfusion fixed with 10% phosphate-buffered formalin at day 7. Slices (10 µm thick) were cut using a microtome (Leica Jung RM2035, Wetzlar, Germany). Slides were dewaxed in xylene and rehydrated in decreasing concentrations of ethanol. Slides were washed in 0.1 mol/L phosphate buffered saline (PBS). Antigen retrieval was performed using the citrate buffer boil method followed by incubation in 1% H_2_0_2_ in methanol for NeuN, Iba1, CNPase and ki-67 and PBS for Olig2. Blocking was performed in 3% normal horse serum (NHS) for NeuN and Iba1 and normal goat serum (NGS) for Olig2, CNPase and ki-67 for 1 hour at room temperature. Sections were labelled with 1∶400 mouse anti-neuronal nuclei monoclonal antibody (NeuN, Chemicon International, Temecula, CA, USA) 1∶400 Olig2 (Chemicon International, Olig2 is a marker for oligodendrocytes at all stages of the lineage, [22]), 1∶200 ki-67 (Dako, Aus), 1∶200 goat anti-Iba1 (Abcam Ltd., Sapphire Bioscience (NZ) Ltd. Hamilton, New Zealand) and 1∶200 mouse anti-CNPase (Abcam) overnight at 4°C. Sections were incubated in biotin-conjugated secondary 1∶200 horse anti-mouse (NeuN) or 1∶200 goat anti-rabbit IgG (Vector Laboratories, Burlingame, USA) in 3.5% NHS. Slides were then incubated in ExtrAvidin (1∶200, Sigma-Aldrich Pty. Ltd, St Louis, USA.) in PBS for two hours at room temperature and then reacted in diaminobenzidine tetrachloride (DAB, Sigma-Aldrich Pty. Ltd.). The reaction was stopped by washing in dH_2_O, the sections dehydrated and mounted. For fluorescent double labelling, dewaxing, rehydrating and antigen retrieval was performed as described above. Sections were blocked in 3% NGS for one hour at room temperature. Sections were incubated with 1∶400 rabbit anti-Olig2 and 1∶200 mouse anti-ki-67 in 3% NGS at 4°C overnight. Sections were washed in PBS and incubated with 1∶200 biotinylated goat anti-mouse IgG (Vector Laboratories) for three hours at room temperature. Sections were washed and incubated with 1∶200 streptavidin Alexa 488 and 1∶200 donkey anti-rabbit Alexa 568.

For all antibodies, two slides per animal were used. To quantify neuronal number, four images in the cortex of the first parasagittal gyrus and one image in both the CA1 and CA3 regions of the hippocampus, were obtained using light microscopy (Nikon Eclipse 80i, Tokyo, Japan). This Neuronal counts were obtained using automated counting software (NIS Elements version 4.0, Nikon). To quantify oligodendrocyte number, one image was obtained in intragyral white matter of both the first and second parasagittal gyrus and one in the periventricular white matter and quantified in the same way by an investigator who was masked to the treatment group. Confocal microscopy was performed on an Olympus Fluoview FV1000 (Image capture: FV10-ASW software, Olympus Corp., Tokyo, Japan).

Brain regions of the forebrain used for analysis included the mid-striatum (comprising the caudate nucleus and putamen), and the frontal subcortical white matter (comprising the intragyral and periventricular regions). The cornu ammonis (CA) of the dorsal horn of the anterior hippocampus (divided into CA1/2, CA3, CA4, and dentate gyrus (DG)) were assessed on sections taken 17 mm anterior to stereotaxic zero. Neuronal (NeuN), oligodendrocyte (Olig-2, CNPase) and microglial (Iba-1) changes, and proliferation (Ki-67) were scored on stained sections by light microscopy at x40 magnification on a Nikon 80i microscope with a motorized stage and Stereo investigator software V.8 (Microbrightfield Inc; Williston, VT, USA) using seven fields in the striatum (four in caudate nucleus, three in putamen), two fields in the white matter (one intragyral, one periventricular) and one field in each of the hippocampal divisions. For each animal, average scores from one section across both hemispheres were calculated for each region.

### Data Analysis

Data was analyzed using ANOVA or repeated measures ANOVA, followed by the Tukey post-hoc test when a significant difference was found. Statistical significance was accepted when p<0.05.

## Results

There were no significant differences in baseline blood gas, pH, glucose or lactate values between the occlusion-vehicle and occlusion-peptide groups (Table 1). Occlusion was associated with severe metabolic and respiratory acidosis in both groups (p<0.05 compared to baseline). There was no significant difference in any parameter during asphyxia or the recovery period between groups.

**Table 1:** Blood gas, pH, glucose and lactate measurements before, during and after 25-vehicle and occlusion-peptide groups.

pH	Baseline	5 min	17 min	+10 min	+1 hour	+2 hour	+4 hour	+6 hour	Day 1	Day 3	Day 7
Occlusion-vehicle	7.39±0.01	7.03±0.02*	6.83±0.01*	7.16±0.01*	7.30±0.01*	7.35±0.01	7.42±0.01	7.41±0.01	7.38±0.01	7.40±0.01	7.39±0.01
Occlusion-peptide	7.39±0.01	7.07±0.02*	6.88±0.01*	7.19±0.01*	7.30±0.01	7.38±0.01	7.43±0.01*	7.41±0.01	7.38±0.02	7.38±0.01	7.37±0.00
**paCO2**											
Occlusion-vehicle	48.1±0.7	107±5.0*	142.1±5.1*	54.6±2.1	42.5±0.9*	43.7±1.3*	43.0±0.6*	47.3±0.7	46.9±0.5	47.1±0.8	48.6±1.7
Occlusion-peptide	48.3±1.3	93.9±3.1*	123.7±0.5*	49.8±1.3	46.5±0.5	47.1±0.4	45.1±0.7	45.0±0.7	43.1±1.3	45.9±0.6	49.8±1.4
**PaO2**											
Occlusion-vehicle	24.7±1.5	6.6±0.6*	7.1±0.8*	34.0±1.5*	30.7±1.2*	26.6±1.6	27.7±1.3	26.6±1.8	27.6±1.0*	27.9±1.0	27.0±1.7
Occlusion-peptide	25.3±1.0	6.9±0.6*	12.4±0.9*	34.8±0.9*	30.8±1.8	26.2±1.5	26.9±1.1	26.9±1.2	29.1±0.9	30.0±1.4	27.2±1.2
**Lactate**											
Occlusion-vehicle	0.9±0.4	4.4±0.2*	6.6±0.5*	6.2±0.4*	4.2±0.1*	3.1±0.4*	2.0±0.3	2.0±0.2*	1.1±0.1	1.0±0.1	0.8±0.1
Occlusion-peptide	0.8±0.0	4.0±0.2*	6.2±0.6*	6.1±0.3*	4.4±0.4*	3.0±0.4*	1.7±0.3	1.8±0.3	1.3±0.2	0.9±0.1	0.7±0.0
**Glucose**											
Occlusion-vehicle	1.0±0.1	0.4±0.1*	0.8±0.1	1.8±0.2*	1.3±0.1	1.4±0.2	1.3±0.2	1.5±0.1*	1.2±0.1	1.1±0.1	1.1±0.1
Occlusion-peptide	1.0±0.1	0.3±0.0*	0.6±0.1*	1.5±0.1*	1.3±0.1*	1.3±0.1*	1.3±0.1*	1.4±0.1*	1.4±0.2	1.2±0.1	1.0±0.1

During asphyxia, there was a significant reduction in pH, PaO_2_ and glucose and a significant increase in PaCO_2_ and lactate in both groups compared to baseline (p<0.05). There was no significant difference in any parameter during asphyxia or the recovery period between groups. Days 2, 4, 5 & 6 were omitted as there were no differences between groups.

**Figure 1:**
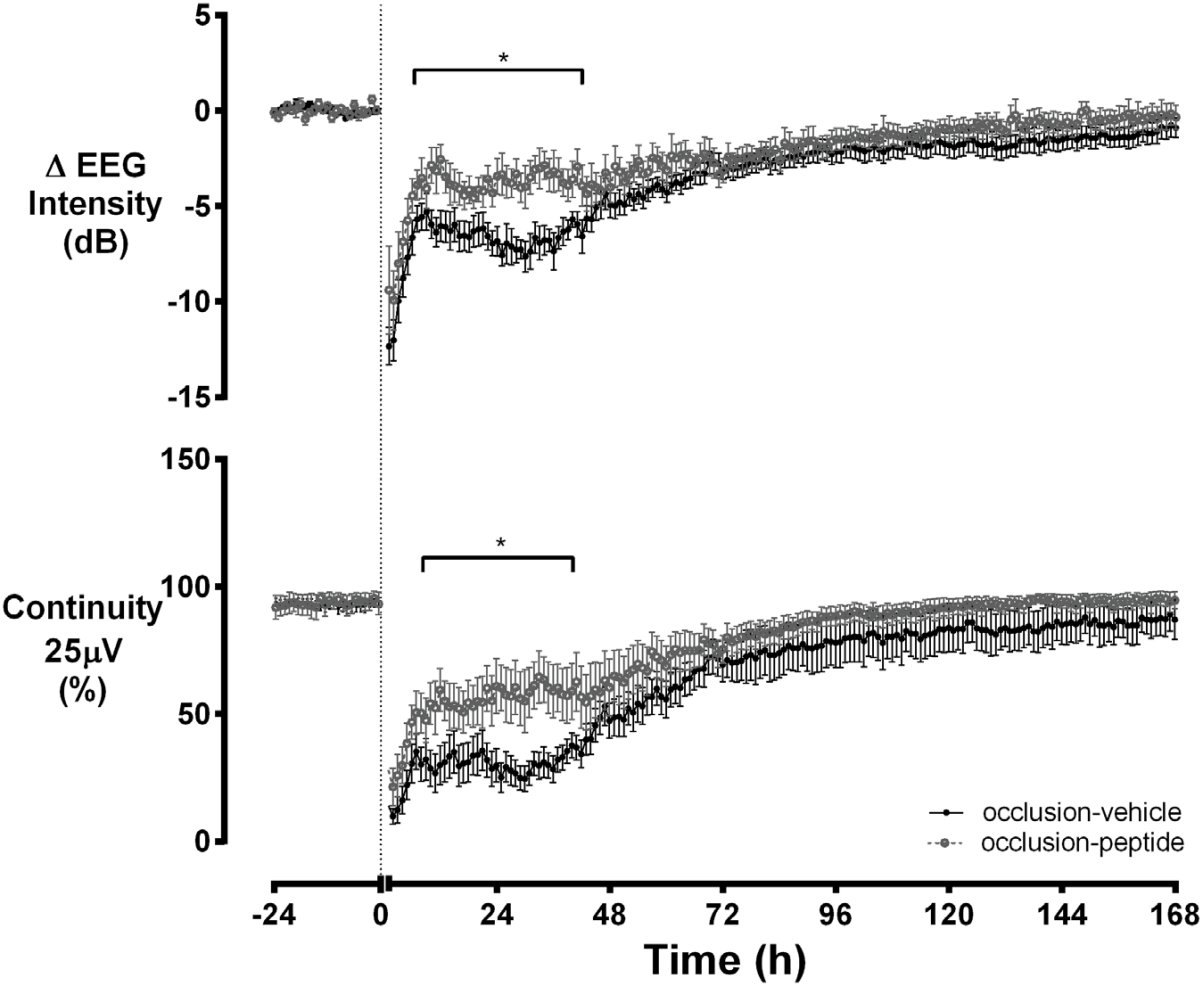
The time sequence of changes in EEG power and continuity at 25 µV during and after 25 min of umbilical cord occlusion. Time point zero denotes the start of occlusion in the occlusion-vehicle and occlusion-peptide groups. EEG activity was suppressed in both groups after asphyxia. EEG power was reduced below baseline until approximately 72 hours after occlusion in the occlusion-vehicle group but was significantly higher between 4–42 hours in the occlusion-peptide group (*p*<0.05). Continuity at 25 µV in the occlusion-peptide group was significantly higher between 4–36 hours compared to the occlusion-vehicle group (*p*<0.05). Data are mean ± SEM.

**Figure 2:**
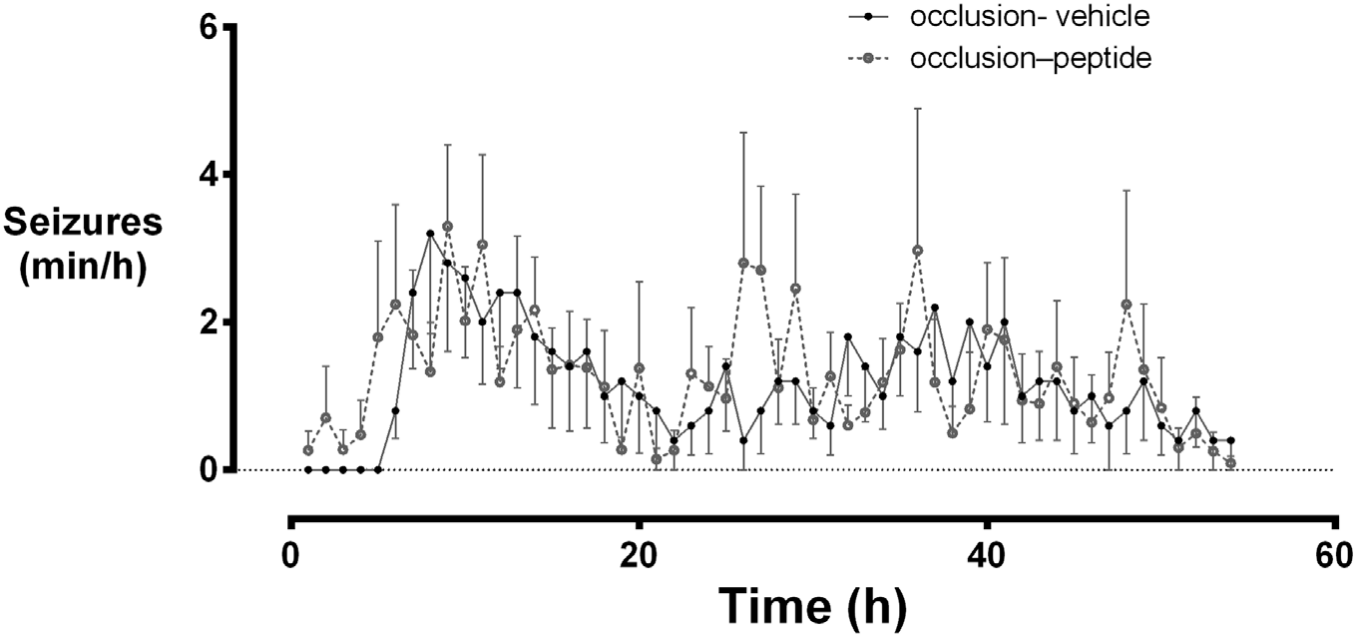
The time sequence of changes in seizure activity (min/hour) in the occlusion-vehicle and occlusion-peptide groups after 25 min of complete umbilical cord occlusion. The maximum seizure burden peaked approximately 8 to 10 hours after the end of occlusion in both groups, with no significant difference in the time spent having seizures.

EEG activity was suppressed below baseline after the end of asphyxia (Figure 1). EEG power gradually returned to baseline in the occlusion-vehicle group. Occlusion-peptide was associated with earlier recovery, with greater EEG power than occlusion-vehicle from 4 to 42 hours (p<0.05). Continuity of the EEG at 25 µV was reduced after the occlusion and returned to baseline significantly earlier in the occlusion-peptide group compared to occlusion-vehicle, with greater continuity from 4 to 36 hours (*p*<0.05). There was no significant difference in seizure burden between groups (Figure 2).

**Figure 3:**
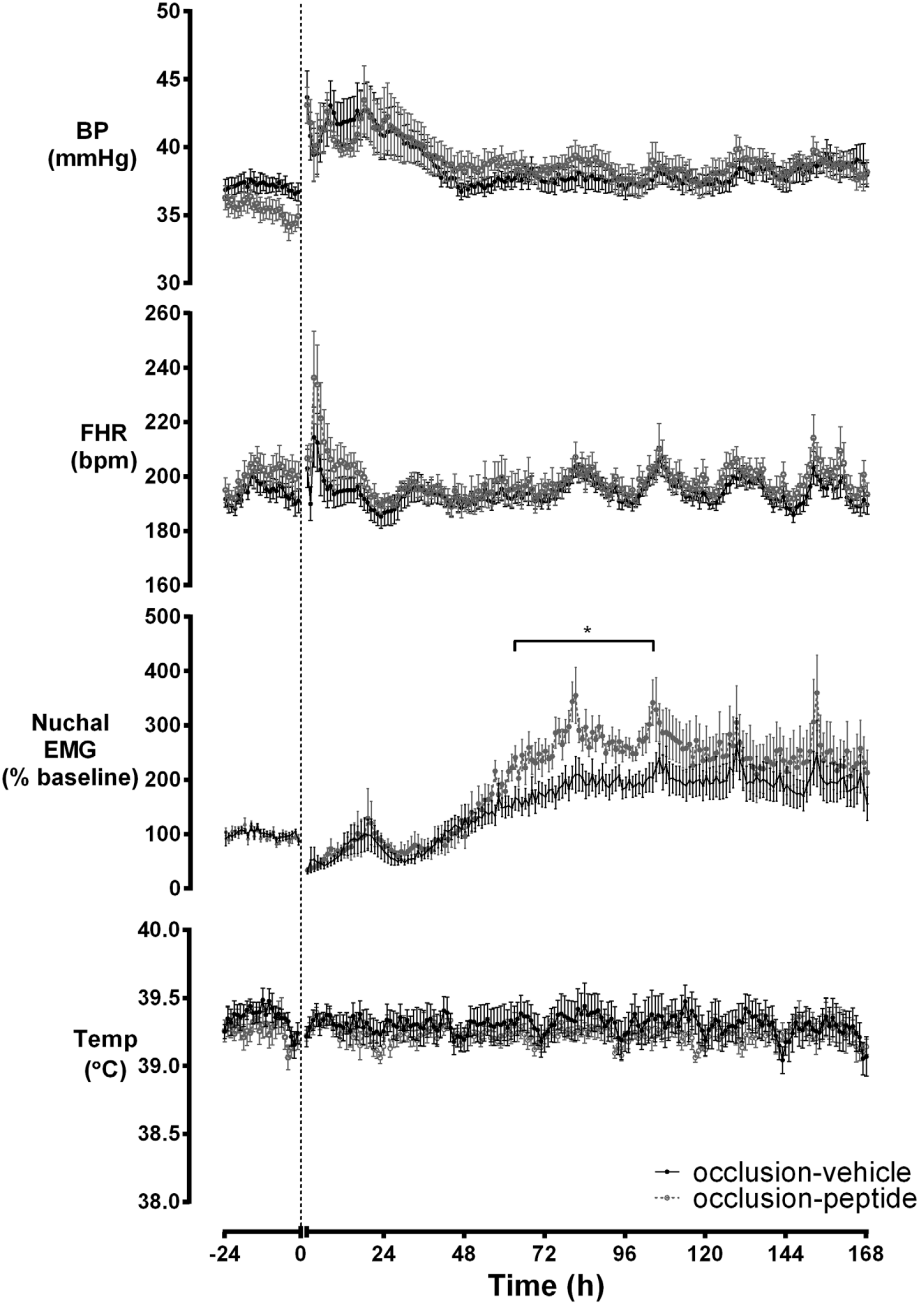
The time sequence of changes in fetal blood pressure, fetal heart rate, nuchal EMG and extradural temperature before and after 25 min of complete umbilical cord occlusion. BP was significantly elevated in both groups after occlusion but returned to baseline by 48 hours. A transient tachycardia was seen in both groups after occlusion. A transient suppression of nuchal EMG was seen after occlusion in both groups followed by an increase to above baseline levels for the remainder of the experiment in both groups that was significantly greater between 62–106 hours in the occlusion-peptide group (p<0.05). No significant differences were seen in extradural temperature between groups.

**Figure 4:**
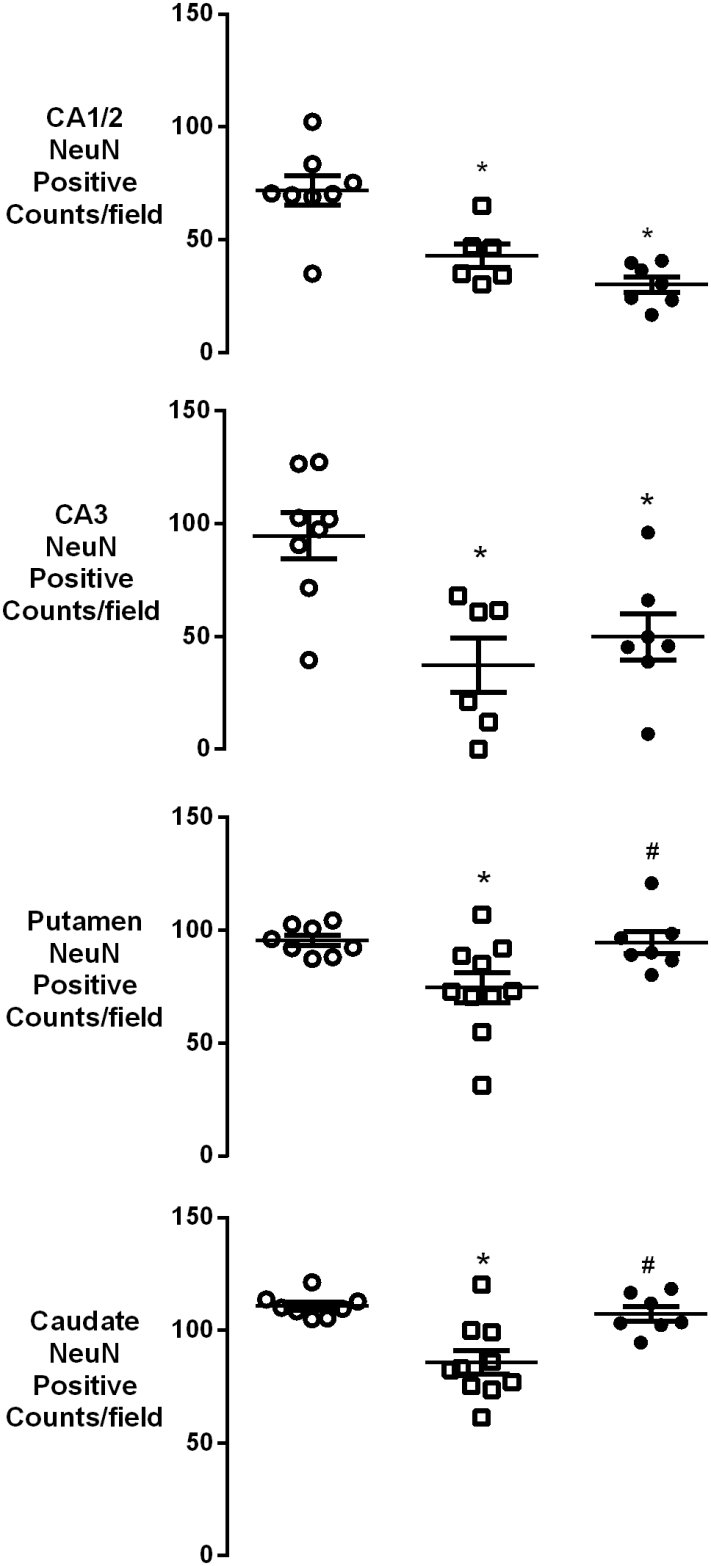
Neuronal cell counts (NeuN) in the hippocampus and striatum seven days after 25 min of complete umbilical cord occlusion. A significant reduction in neuronal cell number was seen in the CA1/2 and CA3 of the hippocampus as well as the caudate and putamen of the striatum in both groups compared to sham control (*p*<0.05). No difference was seen in the CA4 or dentate gyrus compared to sham control. Neuronal cell number was significantly increased in the caudate and putamen in the occlusion-peptide group compared to the occlusion-vehicle group (*p*<0.05). *p<0.05 compared to sham control. #*p*<0.05 compared to ischemia-vehicle. Data are mean ± SEM.

**Figure 5:**
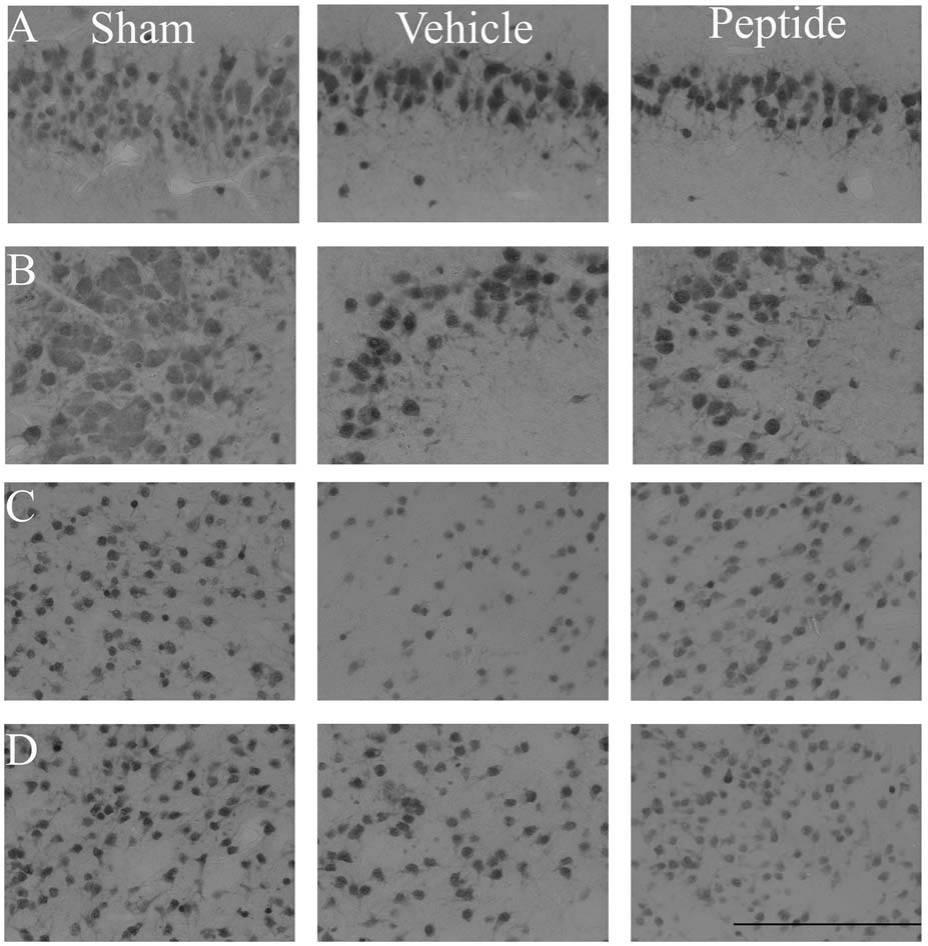
Photomicrographs showing example images of NeuN stained sections in the striatum and hippocampus. Images are shown from sham control (left), occlusion-vehicle (middle) and occlusion-peptide (right) groups in the CA1/2 (panel A), CA3 (panel B), caudate (panel C) and putamen (panel D). Scale bar 200 µm.

Asphyxia was associated with a significant reduction in neuronal number after 7 days recovery in the CA1/2 and CA3 regions of the hippocampus as well as in the caudate and putamen of the striatum, but not in the CA4 or dentate gyrus (data not shown) in the occlusion-vehicle group compared to sham control (*p*<0.05, Figure 4 and Figure 5). In the occlusion-peptide group, neuronal cell number was also significantly reduced in the CA1/2 and CA3 compared to sham control but was significantly increased in the caudate and putamen compared to occlusion-vehicle (*p*<0.05).

**Figure 6:**
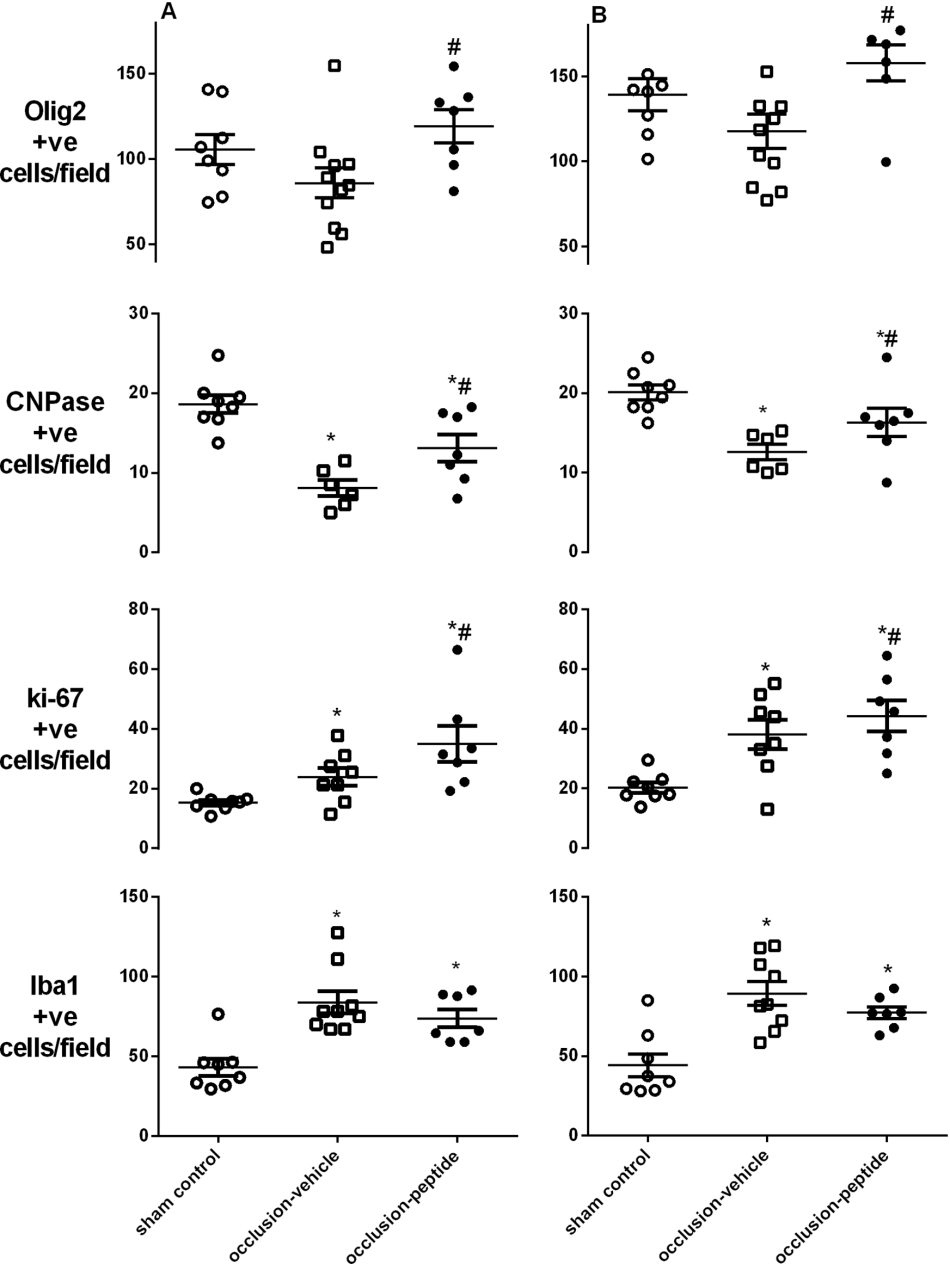
Cell counts of oligodendrocytes, proliferating cells and microglia in the white matter seven days after 25 min of complete umbilical cord occlusion. Cell counts include total oligodendrocytes (Olig-2), immature/mature oligodendrocytes (CNPase), proliferating cells (Ki-67) and total microglial number (Iba-1) in the intragyral (panel A) and periventricular white matter (panel B). A significant increase in total numbers of oligodendrocytes was seen after occlusion-peptide compared to occlusion-vehicle in both the intragyral and periventricular white matter (*p*<0.05). A significant reduction in numbers of immature/mature oligodendrocytes was seen in the occlusion-vehicle group compared to sham control, with an intermediate number in the occlusion-peptide group (*p*<0.05). A significant increase in proliferation was seen in the occlusion-vehicle and occlusion-peptide groups compared to sham control with a further significant increase in the occlusion-peptide group compared to occlusion-vehicle (*p*<0.05). A significant increase in total microglial number was seen in the occlusion-vehicle and occlusion-peptide groups compared to sham control (*p*<0.05). *p<0.05 compared to sham control. #*p*<0.05 compared to the occlusion-vehicle group. Data are mean ± SEM.

**Figure 7:**
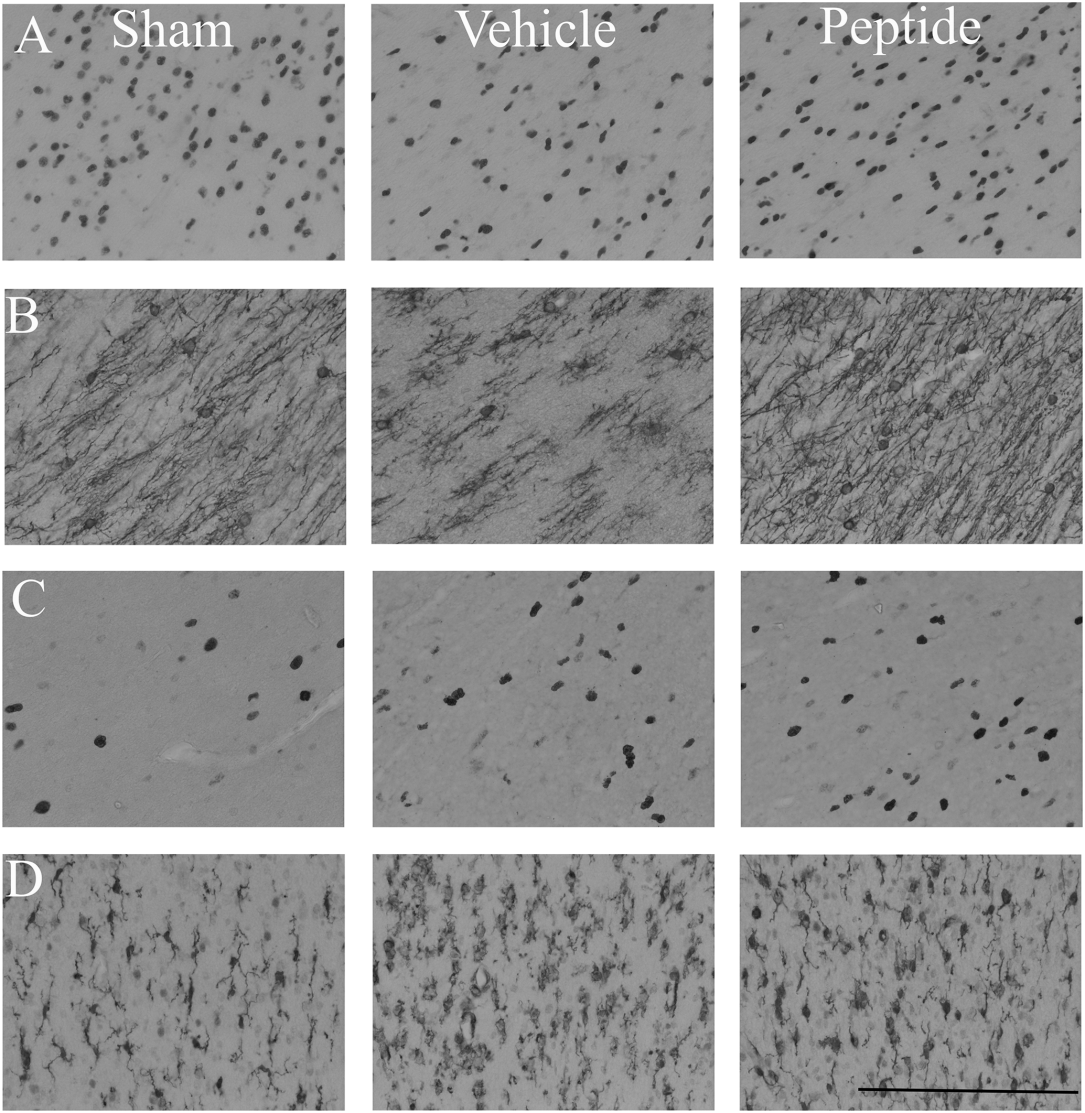
Photomicrographs of oligodendrocytes, proliferating cells and microglia in the white matter seven days after 25 min of complete umbilical cord occlusion. Photomicrographs from the sham control (left column), occlusion-vehicle (middle column) and occlusion-peptide (right column) groups in the intragyral white matter (periventricular white matter not shown). All oligodendrocytes are labelled with Olig-2 (panel A), immature/mature oligodendrocytes with CNPase (panel B), proliferating cells with Ki-67 (panel C) and microglia with Iba-1 (panel D). Scale bar 200 µm.

**Figure 8:**
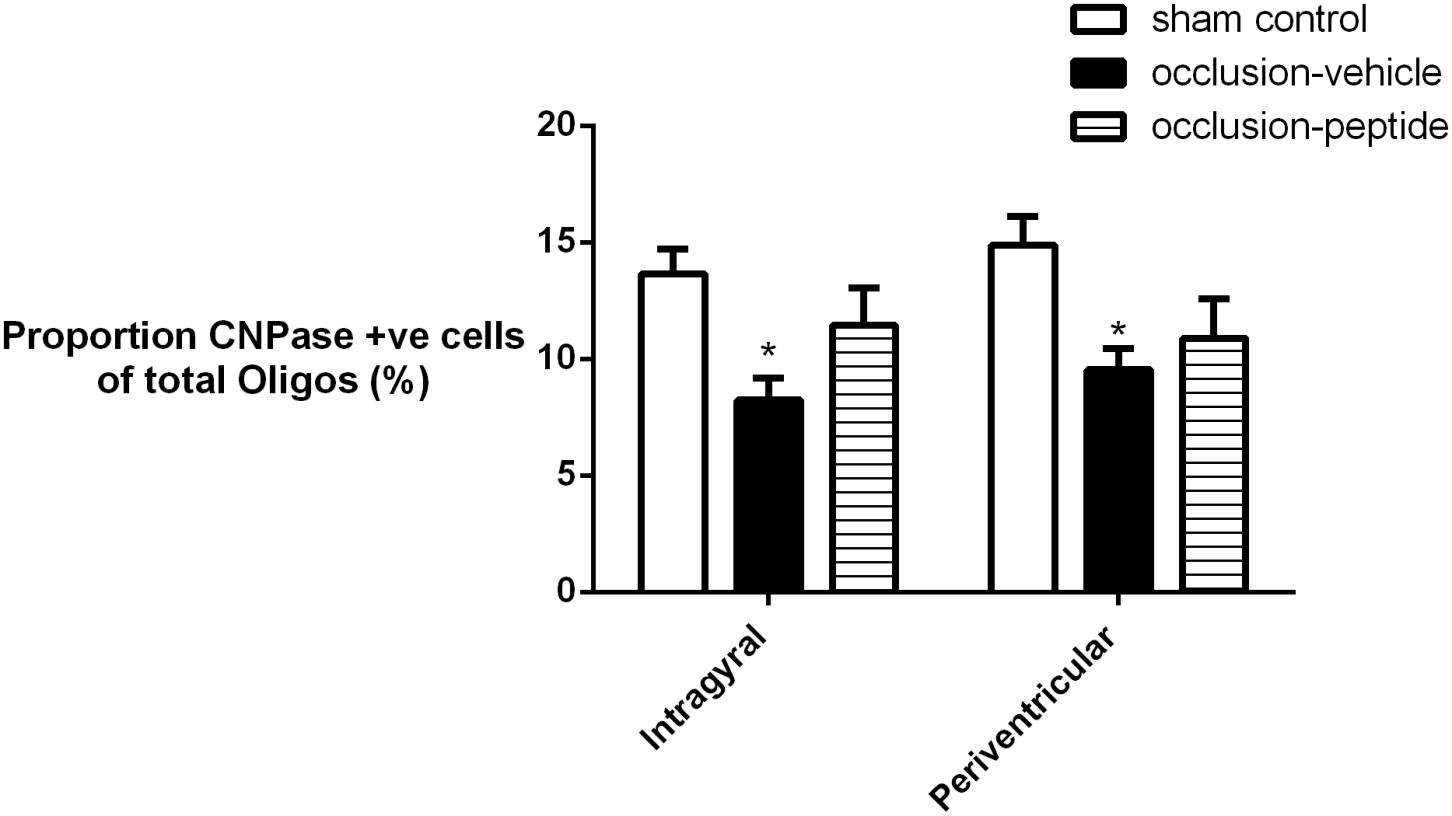
The percentage of CNPase-positive oligodendrocytes seven days after 25 min of complete umbilical cord occlusion. A significant reduction in the percentage of CNPase positive cells relative to total, Olig2-positive oligodendrocytes was seen in the intragyral and periventricular white matter in the occlusion-vehicle group (p<0.05). An intermediate proportion of CNPase positive cells was seen in the occlusion-peptide group, which was not different to sham control or occlusion-vehicle groups in either region.

Asphyxia was associated with a borderline reduction in Olig2-positive oligodendrocytes in both the intragyral and periventricular white matter (Figures 6 and 7). The occlusion-peptide group showed a significant increase in Olig2-positive oligodendrocytes compared to the occlusion-vehicle group (*p*<0.05). Immature/mature (CNPase-positive) oligodendrocytes were significantly reduced in both occlusion groups compared to sham controls in both the intragyral and periventricular white matter (*p*<0.05). Numbers of CNPase-positive oligodendrocytes in the occlusion-peptide group were significantly increased compared to the occlusion-vehicle group (*p*<0.05) in the intragyral and periventricular white matter. The percentage of CNPase positive oligodendrocytes was significantly reduced in the occlusion-vehicle group in both the intragyral and periventricular white matter compared to sham controls (*p*<0.05, Figure 8). The occlusion-peptide group showed an intermediate percentage of CNPase positive oligodendrocytes, and was not significantly different from either sham controls or occlusion-vehicle in both areas.

**Figure 9:**
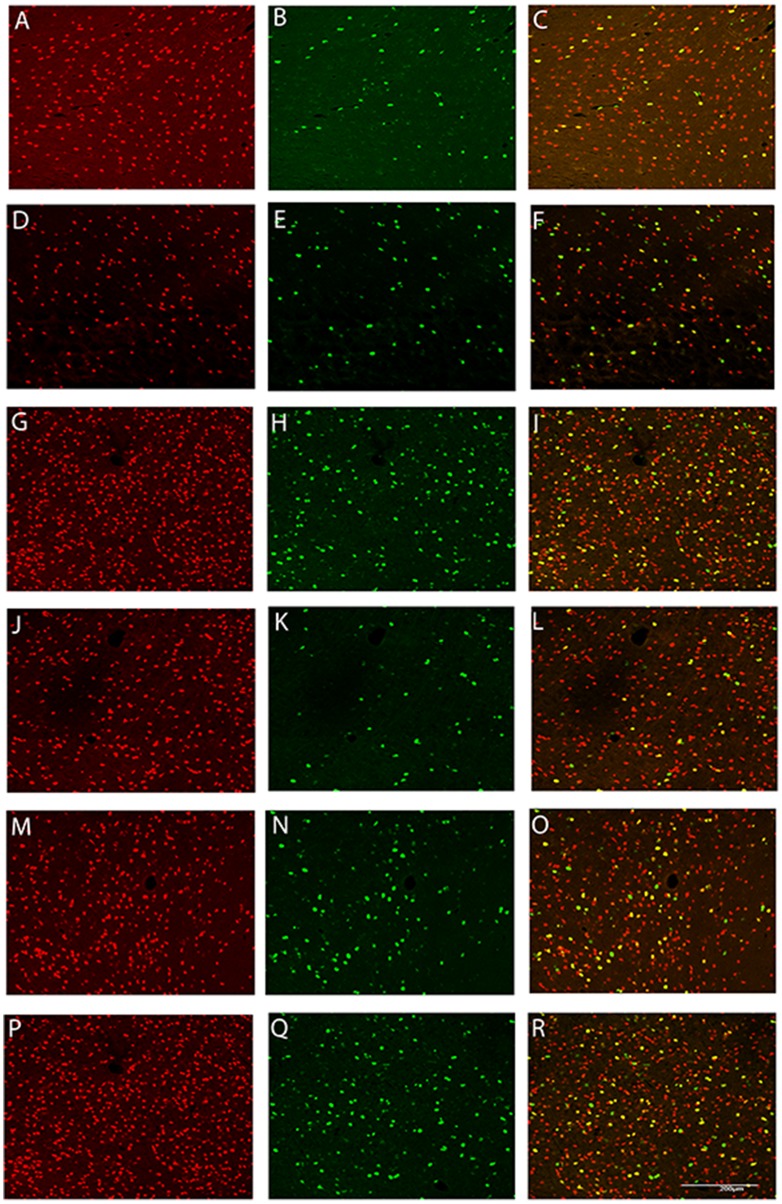
Confocal photomicrographs of double labelled sections for oligodendrocytes and cell proliferation in the white matter seven days after 25 min of complete umbilical cord occlusion. Oligodendrocytes were stained with Olig-2 (left column, red) and proliferating cells were stained with Ki-67 (middle column, green), with colocalized staining of proliferating oligodendrocytes (right column, yellow). Example images are shown from sham control (A–C, J–L), occlusion-vehicle (D–F, M–O) and occlusion-peptide (G–I, P–R) groups in the intragyral (A–I) and periventricular (J–R) white matter. A greater number of proliferating cells and colocalized with oligodendrocytes are seen in both occlusion groups compared to sham control, with the greatest number in the occlusion-peptide group. Scale bar 200 µm.

Asphyxia was associated with a significant increase in proliferating (Ki-67-positive) cells in both the occlusion-vehicle and occlusion-peptide groups compared to sham control in both the intragyral and periventricular white matter (*p*<0.05, Figure 6 and Figure 7). Ki-67 positive cell numbers were further increased in the occlusion-peptide group compared to occlusion-vehicle in the intragyral white matter (*p*<0.05). Confocal microscopy of fluorescent double labeling showed many of the proliferating cells colocalized with oligodendrocytes (Figure 9). Asphyxia was also associated with a significant increase in Iba-1 positive cells (*p*<0.05), with no effect of peptide infusion.

## Discussion

The present study shows for the first time that blockade of connexin 43 hemichannels with a specific mimetic peptide resulted in earlier recovery of brain activity after asphyxia in preterm fetal sheep, associated with a corresponding reduction in neuronal loss in the striatum, enhanced proliferation and improved numbers of immature/mature oligodendrocytes in the white matter tracts. This finding supports the hypothesis that hemichannel opening contributes to spreading hypoxic-ischemic injury even in the very immature brain, at an age that corresponds clinical with the age of greatest vulnerability to neural injury [23].

Acute severe asphyxia was associated with profound suppression of EEG activity, consistent with previous studies [19]. Blockade of connexin hemichannels resulted in earlier recovery of brain activity as seen both by earlier recovery of EEG power to baseline and earlier recovery of EEG continuity compared to the occlusion-vehicle group. Clinically, infants with increased background EEG activity within the first 24 hours after ischemia have better outcome than infants whose background activity remains suppressed [24], [25]. Further, this is consistent with our previous finding in near-term fetal sheep that connexin hemichannel blockade after global cerebral ischemia was associated with both an earlier increase in EEG power and better final recovery of EEG power [13], despite the considerable differences in distribution of injury and particular vulnerability of specific cell types to ischemia between the full-term and preterm neonate [26]–[28].

Subcortical neuronal loss is a known consequence of asphyxia in premature infants and is associated with neurodevelopmental handicap, including cerebral palsy [1], [29]. In the present study, blockade of connexin hemichannels after asphyxia reduced neuronal loss in both the caudate and putamen nuclei of the striatum. However, there was no improvement in neuronal loss in the hippocampus. We can only speculate on potential reasons underlying this lack of efficacy in the hippocampus. The hippocampus is particularly vulnerable to ischemic injury across a range of experimental settings in the fetus [30]–[33]. Injury evolves more quickly with severe injury, and thus the window of opportunity for treatment is less after more severe injury [34]. Alternatively, different mechanisms may contribute to the spread of ischemic brain injury in the hippocampus, or, given that the mimetic peptide was infused directly into the lateral ventricles, it may be that the cells in the hippocampal region were exposed to relatively higher concentrations of mimetic peptide. Higher dose of mimetic peptide are associated with a worse outcome [35], likely mediated by reduced coupling of gap junctions and hence reduced ability of the astrocytic syncytium to maintain homeostasis [21]. We have previously shown intra-cerebroventricular infusion of fluorescent-tagged mimetic peptide in the near-term fetal sheep after global cerebral ischemia is associated with high levels of fluorescence surrounding the ventricles with a graded reduction towards the cortex [35]. Against this hypothesis, in the present study we found improved outcome in the caudate nucleus that is also adjacent to the ventricle and so equally exposed to high peptide concentrations.

In the present study we found no difference in the total number of Olig2 labelled cells in the oligodendrocyte lineage seven days after asphyxia, compared to sham controls. However, there was a significant reduction in CNPase labeled immature/mature oligodendrocytes, as well as a reduction in percentage of CNPase positive cells. Given that the Olig2 labeled cells include all immature/mature oligodendrocytes [22], these data are consistent with the hypothesis that the reduction in immature/mature oligodendrocytes corresponds with increased numbers of preoligodendrocytes. We have previously shown that in the same model as the present study, there is significant loss of oligodendrocyte progenitor cells three days after asphyxia [33], [36] and we now show that after 7 days there was a significant increase in proliferation in the occlusion-vehicle group compared to sham control. The exuberant proliferation response to injury is almost entirely mediated by oligodendrocyte progenitor cells [37], as shown here by co-localization of Olig2 and Ki-67. Taken as a whole, these data suggest early loss of preoligodendrocytes [36] followed by significant proliferation of new preoligodendrocytes with either impaired maturation, or at least failure to replace immature/mature oligodendrocytes by day 7. This is consistent with data in the neonatal rat and the preterm fetal sheep that showed degeneration of preoligodendrocytes offset by dramatic proliferation, but impaired maturation after hypoxia ischemia [27], [28] and with the critical finding of maturational arrest of preoligodendrocytes in human neonatal white matter injury at post-mortem [2].

Strikingly, blockade of connexin hemichannels significantly increased both the total number of oligodendrocytes as well the number of mature oligodendrocytes in the white matter, with a further significant increase in proliferation in the intragyral white matter. The percentage of CNPase positive cells was intermediate between sham controls and occlusion-vehicle, and not significantly different from either group. This suggests that blockade of connexin hemichannels may have reduced oligodendrocyte cell loss, enhanced proliferation of new oligodendrocytes and/or help partially restore maturation of oligodendrocyte lineage development. Intriguingly, blockade of connexin hemichannels has no known direct effect on proliferation. Therefore the increased oligodendrocyte numbers is presumptively mediated indirectly through reduced acute cell death and restoration of a more favorable extracellular environment conducive to cell survival, proliferation and maturation.

Blockade of connexin hemichannels after asphyxia in preterm fetal sheep did not have any significant effect on seizure activity in the present study, in contrast with marked reduction in status epilepticus in the near-term fetal sheep [13]. A key difference between these studies is that in contrast with the common development of status epilepticus after ischemia in the term-equivalent fetal sheep, discrete seizures predominate in the preterm fetus [33], [38]. There is considerable evidence implicating gap junctions and/or connexin hemichannels in the initiation, propagation and particularly in the continuity of seizure activity [39]–[41]. We speculate that connexin hemichannel blockade attenuates the propagation of abnormal electrical activity rather than the generation of seizures, and so had no effect on the discrete seizures seen in this study.

There are also considerable differences in the characteristic patterns of neural injury and the cell types affected in the preterm compared to the term neonate. At term, injury is characterized by profound cortical and subcortical neuronal loss with some white matter injury, whereas preterm brain injury is associated with severe white matter injury, with particular vulnerability of the premyelinating oligodendrocytes and some subcortical neuronal loss, but sparing of cortical neurons [26]–[28]. Despite these differences in the pattern, pathogenesis and specific cell vulnerability to global hypoxic-ischemic brain injury between the near-term and preterm neonate, blockade of connexin hemichannels significantly reduced cell loss and improved recovery of EEG activity at both gestational ages [13]. Further, opening of connexin hemichannels has been implicated in the spread of injury in models of adult ischemic stroke and of retinal ischemia [8], [42], [43]. This suggests that connexin hemichannels are a common mechanism in the spread of ischemic brain injury across a wide range of brain maturity and types of ischemic insults.

Importantly for clinical translation, we have shown in the near-term fetal sheep that connexin hemichannels play a role in the spread of brain injury after ischemia but do not appear to contribute significantly during the period of ischemia itself [44]. Consistent with this, we found that connexin hemichannel mRNA expression is significantly upregulated four hours after the end of ischemia in the near-term fetal sheep [13]. This delay allows time for identification of infants that may potentially benefit from mimetic peptide therapy. Based on this evidence, in the present study peptide infusion was begun after 90 min recovery from asphyxia, in order to model a clinically realistic treatment protocol.

Reassuringly, blockade of connexin hemichannels after asphyxia had no effect on mean arterial pressure, fetal heart rate or extradural temperature. A greater increase in body movements, as measured by nuchal EMG activity, was seen between 62–106 hours in the occlusion-peptide group compared to the occlusion-vehicle group. This may reflect improved early behavioral recovery. We have previously shown that the neuroprotective effects of peptide infusion are specific to the particular mimetic peptide administered in this study as an alternate peptide targeting another region of Cx43 did not affect neural injury after ischemia in near-term fetal sheep [13]. A limitation of the present study is that we did not examine the effect of mimetic peptide infusion in healthy preterm fetal sheep. Reassuringly, in healthy 0.85 gestation fetal sheep, at an age when the fetal sheep neural maturation is consistent with that of the full term human infant [16], we found no effect of mimetic peptide infusion at the same dose per kg as the present study on normal brain activity [13]. Despite continuous long-term monitoring of these animals, we did not observe any off target effects, however, it is not possible to wholly exclude this possibility.

The present study showed for the first time that blockade of connexin hemichannels improved recovery of brain activity as well as subcortical neuronal and white matter cell survival and maturation in the preterm fetal sheep. These data suggest that blockade of connexin hemichannels may be a useful therapeutic intervention for the treatment of preterm infants following asphyxia.
